# Interpreting cerebrospinal fluid pleocytosis in HIV in the era of potent antiretroviral therapy

**DOI:** 10.1186/1471-2334-7-37

**Published:** 2007-05-02

**Authors:** Christina M Marra, Clare L Maxwell, Ann C Collier, Kevin R Robertson, Allison Imrie

**Affiliations:** 1 Harborview Medical Center, University of Washington, Seattle, WA, USA; 2 Department of Neurology, University of Washington, Seattle, WA, USA; 3 Department of Neurology, University of North Carolina School of Medicine, Chapel Hill, NC, USA; 4 Department of Public Health Sciences, University of Hawaii at Manoa John A. Burns School of Medicine, Honolulu, HI, USA

## Abstract

**Background:**

Cerebrospinal fluid (CSF) pleocytosis may be seen in asymptomatic HIV-infected individuals. This finding complicates interpretation of CSF abnormalities when such individuals are evaluated for other central nervous system infections. The goal of this study was to determine the relationship between CSF pleocytosis, central nervous system (CNS) antiretroviral penetration, adherence to antiretroviral medication regimens, neurological symptoms and performance on neuropsychological tests.

**Methods:**

Clinically stable HIV-infected individuals at any peripheral blood CD4+ T cell count or any plasma viral load were asked to attend study visits at entry and every 6 months thereafter for at least one year. At each visit, they underwent a standardized neurological and medication history; neurological examination; a brief neuropsychological test battery: venipuncture; lumbar puncture; and assessment of medication adherence. Generalized estimating equations (GEE) were used to assess the relationships between CSF pleocytosis and other variables.

**Results:**

CSF pleocytosis was independently and significantly related to lack of current antiretroviral use (OR 5.9, 95% CI 1.8–18.6, p = 0.003), CD4 count > 200/ul (OR 23.4, 95% CI 3.1–177.3, p = 0.002) and detectable plasma HIV RNA (OR 3.3, 95% CI 1.1–9.4, p = 0.03). At visits where antiretrovirals were used, and taking into account detectable plasma HIV RNA, an antiretroviral regimen that contained two or more agents with good CNS penetration conferred a trend toward lower odds of CSF pleocytosis (OR 0.45, 95% CI 0.18–1.12, p = 0.087).

**Conclusion:**

CSF pleocytosis is a characteristic of HIV disease that varies significantly with easily identifiable clinical and laboratory features. Use of antiretroviral agents decreases the odds of pleocytosis. This association may be stronger when the regimen contains two or more agents with good CNS penetration.

## Background

Cerebrospinal fluid (CSF) pleocytosis is common in HIV-infected individuals who do not have other central nervous system infections (CNS), and it is not accompanied by neurological symptoms or abnormalities on neuropsychological tests [1-4]. Several studies show that CSF HIV RNA concentration increases with increasing CSF white blood cells (WBC) [4-6]. CSF pleocytosis decreases after antiretroviral treatment, even when the regimen is only partially effective, and treatment interruption may lead to increases in CSF WBC [1,7]. Most studies have focused on correlates of CSF viral replication, rather than on CSF pleocytosis. A better understanding of the factors that influence HIV-related CSF pleocytosis would assist in interpretation of CSF abnormalities when CNS infection other than HIV is suspected. The goal of this study was to determine the relationship between CSF pleocytosis, CNS antiretroviral penetration, adherence to antiretroviral medication regimens and performance on neuropsychological tests. Our results show that CSF pleocytosis is a characteristic of HIV disease that varies significantly with easily identifiable clinical and laboratory features.

## Methods

After written consent was obtained, clinically stable HIV-infected individuals with any peripheral blood CD4 count or any plasma viral load were asked to attend study visits at entry and every 6 months thereafter for at least one year. At each visit, they underwent a standardized neurological and medication history; neurological examination; brief neuropsychological test battery that included timed gait, grooved pegboard with the dominant hand, finger tapping with the nondominant hand and digit symbol; assessment of antiretroviral drug adherence [8]; venipuncture; and lumbar puncture. Adherence was defined as taking ≥ 95% of prescribed doses in the 4 days before each visit on a standardized self-reported questionnaire. Based on review of medical records, and on medical history and physical examination, no subject had an intercurrent illness other than HIV. No subject underwent lumbar puncture for clinical indications. The study protocol was reviewed and approved by the University of Washington Institutional Review Board. Human experimentation guidelines of the University of Washington were followed in the conduct of this research.

Cerebrospinal fluid WBCs and HIV RNA were considered as dichotomous variables: CSF pleocytosis was defined as CSF WBC > 5/ul and plasma and CSF samples with HIV RNA ≤ 50 copies/ml were considered undetectable. This approach was justified for two related reasons. First, these variables show dichotomous distributions (normal or undetectable vs. elevated or detectable) mixed with skewed continuous distributions for values above the limits of normal or detectable. This precludes the use of any simple regression model. Second, the presence or absence of pleocytosis or detectability of HIV RNA are more clinically relevant to our analysis than the magnitude of the abnormal values. As in other studies, we defined drugs with good CNS antiretroviral penetration as zidovudine, abacavir, stavudine, nevirapine, efavirenz, lamivudine and indinavir [9]. We categorized regimens containing none or one of these agents as poorly penetrant regimens and those containing two or more of these agents as penetrant regimens. Z scores were calculated for each neuropsychological test using age-adjusted norms [10], and a composite Z score (NPZ4) was calculated at each visit. Values greater than 3 standard deviations above or below the mean were considered outliers (two scores of less than -3.0 standard deviations from two different subjects) and were excluded. Baseline associations were determined using Fisher's exact or chi-square tests. Generalized estimating equations (GEE) were used to assess the relationship between variables of interest over all study visits, which allowed us to include data from subjects who changed their antiretroviral regimen, account for correlation between same-subject visits, examine potential time trends and control for these if necessary. All tests were two-tailed and p values ≤ 0.05 were considered significant.

## Results

Fifty subjects were enrolled and underwent a total of 143 visits (median 3, range 1–6). Median study participation was 11.9 months (range 0–30). Subject characteristics at entry are shown in Table 1; their demographic characteristics are representative of our hospital's HIV clinic attendees during the same time period (personal communication, Mari Kitahata, MD, 02/23/2006). Of the 33 subjects taking antiretrovirals at entry, the median number of agents was 3 (range, 2–5). During the study, 18 of 33 subjects made changes in their antiviral regimen. Four subjects who entered the study on antiretrovirals were not taking them at one study visit, and two subjects initiated treatment. One subject who stopped antiretrovirals and then started them again had an increase in CSF WBCs from 7/ul to 218/ul, suggesting an immune reconstitution syndrome. No subject had intercurrent CNS infection apart from HIV.

**Table 1:** Subject Characteristics at the Entry Visit

Characteristic	Value
Age, years, median (range)	41.5 (23–64)
Male, n (%)	39 (78%)
Race, n (%) White, not Hispanic	33 (66%)
Black, not Hispanic	10 (20%)
Hispanic	4 (8%)
Other	3 (6%)
Years of education, median (range)	13 (9–18)
NPZ4, mean (standard deviation)	-0.46 (0.72)
Current antiretroviral use, n (%)	33 (66%)
ARVs with good CNS penetration, median (range)	3 (0–4)
Two or more ARVs with good CNS penetration, n (%)	28 (85%)
Peripheral blood CD4+ T cells/ul, median (range)	467 (3–1016)
Plasma HIV RNA (49 subjects)	
Detectable n (%)	29 (58%)
RNA log copies/ml in those with detectable plasma HIV RNA, median (range)	3.50 (1.82–5.48)
CSF WBC	
WBC > 5/ul, n (%)	15 (30%)
CSF WBC in those with > 5/ul, median, (range)	13 (7–51)
CSF HIV RNA	
Detectable, n (%)	17 (34%)
CSF HIV RNA log copies/ml in those with detectable CSF HIV RNA, median (range)	2.92 (1.71–4.08)

HIV, human immunodeficiency virus

NPZ4, normalized score on four neuropsychological tests

RNA, ribonucleic acid

CSF, cerebrospinal fluid

WBC, white blood cell

Detectable, > 50 copies/ml

ARV, antiretroviral agent

At entry, CSF pleocytosis was significantly more likely in subjects not taking antiretrovirals (p = 0.001); and with peripheral blood CD4+ T cells > 200/ul (p = 0.04), detectable plasma HIV RNA (p = 0.05) and detectable CSF HIV RNA (p = 0.001). In a multivariate model including all visits, CSF pleocytosis remained independently and significantly related to lack of current antiretroviral use, peripheral blood CD4+ T cells > 200/ul and detectable plasma HIV RNA, but not to detectable CSF HIV RNA (Table 2). There was no relationship between CSF pleocytosis and medication adherence (data not shown). At visits where antiretrovirals were used, and taking into account detectable plasma HIV RNA, an antiretroviral regimen that contained two or more agents with good CNS penetration conferred a trend toward lower odds of CSF pleocytosis (OR 0.45, 95% CI 0.18–1.12, p = 0.087). There were no significant time trends.

**Table 2:** Significant predictors of CSF WBC > 5/ul

	Adjusted OR (95% CI)	P-value
No current use of antiretroviral medications	5.9 (1.8–18.6)	0.003
Peripheral blood CD4+ T cells > 200/ul	23.4 (3.1–177.3)	0.002
Detectable plasma HIV RNA	3.3 (1.1–9.4)	0.03

Detectable, > 50 copies/ml

Odds ratios are adjusted for the presence of the other two risk factors.

The most common neurological symptoms at entry were mild or moderate difficulty concentrating in 15 (30%) subjects and mild or moderate memory difficulty in 22 (44%) subjects. Over the course of the study, CSF pleocytosis was no more common in subjects with or without these symptoms. There were no significant time trends. Similarly, CSF pleocytosis was not related to neuropsychological test performance when results were considered as a continuous variable or dichotomized as clinically impaired (NPZ4 ≤ -0.5) vs. not impaired (NPZ4 > -0.5) (data not shown). In this analysis, we controlled for improved performance on the neuropsychological test battery at sequential visits, which may reflect a practice effect.

## Discussion

HIV can cause mild CSF pleocytosis. Indeed, at the baseline visit, 30% of our subjects had pleocytosis. We show that across all visits, lack of use of antiretroviral agents, CD4 count > 200/ul and detectable plasma viral load independently and significantly increase the odds of CSF pleocytosis, presumably due to HIV itself. Although CSF viral load was associated with CSF pleocytosis in bivariate analysis, the association was lost in multivariate analysis. At visits where antiretrovirals were used, and taking into account detectable plasma HIV RNA, an antiretroviral regimen that contained two or more agents with good CNS penetration conferred a trend toward lower odds of CSF pleocytosis. The relationships that we observed between CSF pleocytosis, antiretroviral use and CNS penetration of a given regimen support the contention that CSF pleocytosis is a direct reflection of CSF HIV infection.

The focus of our study differs from others because we addressed factors that predict CSF pleocytosis rather than the influence of CSF pleocytosis on CSF HIV replication. Our results extend previous observations. Specifically, we provide quantitative estimates of the odds of CSF pleocytosis based on clinical features that are easy to identify: antiretroviral use, CD4 count and plasma HIV RNA. Because each of these three variables is significantly and independently associated with CSF pleocytosis, multiplying the odds ratios of these covariates for an individual patient can provide an overall estimate of the odds of CSF pleocytosis due to HIV infection. These findings have direct clinical relevance to the situation of an HIV-infected patient with suspected CSF or CNS infection and CSF pleocyosis. When the overall odds of HIV-related CSF pleocytosis are low, as they would be for an individual with advanced HIV infection currently on antiretroviral therapy with suppressed plasma viremia, an explanation for CSF pleocytosis other than HIV should be sought.

In contrast to our results, a recent cross-sectional analysis identified only a weak correlation between CSF WBCs and CD4 count, and found no relationship between CSF pleocytosis and plasma viral load [1]. In agreement with their results, we found no relationship between cognitive complaints or NPZ4 in subjects with or without CSF pleocytosis. Differences between study results may be due to several factors. Our subjects had less advanced HIV disease. Because CSF WBCs, our dependent variable, shows a mixed distribution, it could not be modeled as a continuous outcome. Thus we used the dichotomous component of this variable because of its clear clinical relevance, and we measured the contributions of peripheral viral load, CD4 count and antiretroviral therapy to pleocytosis jointly, rather than individually.

Limitations of our study should be noted. Our sample size was modest, limiting power to detect some associations. However, using GEE models allowed us to analyze data from all 143 subject visits, and we identified several significant associations. Ours was a convenience sample, but our subjects demographically and clinically resembled our HIV clinic population, suggesting that our results may be generalizable to HIV-infected patients in clinical care in similar communities. Subjects did not have intercurrent illnesses based on record review, history and clinical examination. It is possible that we could have misclassified asymptomatic meningitis as due to HIV when it might have been due to some other process. However, such misclassification would likely have weakened, rather than strengthened, our ability to identify associations between CSF pleocytosis, peripheral blood CD4+ T cells, plasma HIV RNA and use of potent antiretrovirals.

## Conclusion

CSF pleocytosis is a characteristic of HIV disease that varies significantly with easily identifiable clinical and laboratory features. Knowledge of these factors is essential in interpreting the CSF profile of such individuals when they are evaluated for CNS infections other than HIV, such as meningitis or encephalitis. Our data provide a practical approach to address this issue.

## Competing interests

The author(s) declare that they have no competing interests.

## Authors' contributions

CMM participated in conception and design of the study, acquisition of data, analysis and interpretation of data, drafting of manuscript, critical revision of manuscript for important intellectual content, and obtained funding. CLM participated in analysis and interpretation of data, drafting of manuscript, critical revision of manuscript for important intellectual content, and provided statistical expertise. AC participated in conception and design of the study, critical revision of the manuscript for important intellectual content, and provided administrative, technical or material support. KR participated in conception and design of the study, analysis and interpretation of data, critical revision of manuscript for important intellectual content, and provided administrative, technical or material support. AI participated in conception and design, analysis and interpretation of data, critical revision of manuscript for important intellectual content, and obtained funding. All authors read and approved the final manuscript.

## Pre-publication history

The pre-publication history for this paper can be accessed here:


